# Identification of repurposable drugs with beneficial effects on glucose control in type 2 diabetes using machine learning

**DOI:** 10.1002/prp2.529

**Published:** 2019-11-20

**Authors:** Gideon Koren, Galia Nordon, Kira Radinsky, Varda Shalev

**Affiliations:** ^1^ Ariel University Ariel Israel; ^2^ Maccabi Institute for Research and Innovation Tel Aviv Israel; ^3^ Technion The Israeli Institute for Technology Haifa Israel

**Keywords:** big data analysis, diabetes type 2, glucose control, machine learning, α1‐adrenoceptor antagonist

## Abstract

Despite effective medications, rates of uncontrolled glucose levels in type 2 diabetes remain high. We aimed to test the utility of machine learning applied to big data in identifying the potential role of concomitant drugs not taken for diabetes which may contribute to lowering blood glucose. Success in controlling blood glucose was defined as achieving HgA1c levels < 6.5% after 90‐365 days following diagnosis and initiating treatment. Among numerous concomitant drugs taken by type 2 diabetic patients, alpha 1 (α1)‐adrenoceptor antagonist drugs were the only group of medications that significantly improved the success rate of glucose control. Searching the published literature, this effect of α1‐adrenoceptor antagonists has been shown in animal models, where this class of medications appears to induce insulin secretion. In conclusion, machine learning of big data is a novel method to identify effective antidiabetic effects for potential repurposable medications already on the market for other indications. Because these α1‐adrenoceptor antagonists are widely used in men for treating benign prostate hyperplasia (BPH) at age groups exhibiting increased rates of type 2 diabetes, this finding is of potential clinical significance.

**Abbreviations**
BPH: benign prostate hyperplasia
DM‐2: Diabetes Mellitus type 2
DPP‐4: dipeptidyl peptidase
GLP: glucagon‐like peptide

## 1 INTRODUCTION

Diabetes Mellitus type 2 (DM‐2) is a chronic condition afflicting increasing numbers of individuals worldwide, and adversely affecting their health, quality of life, and survival.^1^

In addition to dietary modifications, exercise and other lifestyle changes, the majority of DM‐2 patients are treated chronically with the several groups of medications.^2^ These include insulin, meglitinides, sulfonylureas, thiazolidinediones, dipeptidyl peptidase (DPP‐4) inhibitors, glucagon‐like peptide (GLP) 1 receptor agonists, and sodium transport protein 2 (SGLT 2) inhibitors. Yet, despite extensive efforts, balancing carbohydrate metabolism remains a challenge for many patients.^1^ In an effort to find new ways to identify medications that can help in balancing DM‐2, we have employed big data machine learning techniques, recently validated by us in successfully identifying repurposable antihypertensive drugs.^3^

We present evidence that alpha 1 (α1)‐adrenoceptor antagonists have a significant favorable effect on balancing DM type 2 when combined with known antidiabetic drugs.

## 2 METHODS

From the electronic medical charts of Maccabi Health Services, the second largest health service organization in Israel insuring over 2 million members,^4^ we identified patients receiving their first‐ever drug treatment for DM‐2 after a diagnosis had been made. Medications utilized were identified from the electronically recorded purchases of the patient. For these patients, initial blood glucose values were recorded before treatment. Weight, age, BMI, and smoking status were extracted from the electronic medical charts, calculating their mean, median, maximum, minimum, and standard deviation. Mean HgA1c levels for these patients were calculated for the period between 90 and 365 days following the date of diagnosis. Patients with HgA1c levels <6.5 were classified as successful treatment and based on this criterion 54% of the patients were successfully treated. The study was approved by Assuta Hospital Research Ethics Committee in Tel Aviv permitting access to the patients’ files.

### 2.1 Machine learning methodology

“Classification” is a task of machine learning in which the data can be divided into separate categories or classes. The algorithm is designed to predict the correct class for each data item in the repository. In our case there were two classes: “treatment success” by achieving HgA1c levels <6.5 within 90‐365 days of treatment initiation, and “treatment failure” (all other cases). We used two types of machine learning algorithms: Decision trees and fully connected neural networks. The analysis was done employing Python. Statistical and machine learning analyses were performed with infrastructure from Scipy, Ssikit‐learn.^5^, ^6^, ^7^, ^8^

We systematically surveyed drug groups and compared HgA1c levels of treated and untreated diabetic patients with antidiabetic drugs. In an attempt to eliminate as much patient variability as possible among the treated and untreated groups, we used propensity score matching to examine whether a specific drug treatment/combination achieved independently higher success rates.^9^, ^10^, ^11^, ^12^. Using this method, we trained a regression model to predict the probability of the patient's treatment success when taking a given drug. The treated and untreated groups were constructed in such a way that the propensity scores of the groups were as similar as possible.

We used the following patient characteristics for the matching: weight, age, BMI, and smoking status. Treatment groups were excluded according to the rate of resampling and Kolmogorov‐Smirnof (KS) goodness‐of‐fit tests for all features.^5^, ^6^, ^7^, ^8^ We chose a resampling rate of 20%, with *P*‐values of less than .0001 for a single feature, as our limit for group's exclusion. Specifically, if the KS test for one of the features we matched had *P*‐value of greater than .001, we considered it to be insufficiently strong to prove treatment success. Resampling was allowed in the matching process (ie, the same patient could be matched to several patients from the original group). The basic concept behind matching is to try to match one group of observations with another group of observations in such a way that the items in the groups are as similar as possible in all aspects except for the tested variable. In our case, given a group of patients treated with drug x, we aimed to match every patient with a patient who was identical to him/her in age, weight, BMI etc except for the fact that the matched patient was not treated with drug x.

We performed an exhaustive search over all treatment groups, excluding drugs that were bought by less than 200 patients and identified 73 such groups. For each treatment group, we compared treatment success rates of the group of patients treated with that specific drug to a matched group of patients who were not treated with that specific treatment. Based on the entire database, logistic regression was used for predicting the probability of treatment success with the matched drug and this constituted the propensity score. For each patient in the treated group we matched a patient untreated with that specific treatment with the closest propensity score.

Pearson's chi‐squared test was used to determine whether the success rates differed among groups. To accommodate for multihypothesis testing, the *P*‐values were corrected according to the Bonferroni correction. We present the five smallest chi square *P*‐values including the Bonferroni corrected *P*.

## 3 RESULTS

### 3.1 Main analysis (Table 1)

We extracted 29 540 patients diagnosed with type 2 diabetes between 2005 and mid‐2016. Mean HgA1c levels for these patients were calculated for the period between 90 and 365 days following diagnosis date. Patients with HgA1c levels <6.5 were classified as successful treatment and 54% of the patients were successfully treated.

**Table 1:** Comparison of success in glucose control between patients with BPH treated and untreated with/without diabetes

	Diabetes	Prostate	Prostate drug	Total patients	Treatment success*	Treatment fail
Group1	+	+	−	253	151 (60%)	102
Group2	+	−	−	246	119 (48%)	127

Note

Out of 26 537 patients diagnosed with BPH there were 5756 patients who did not receive α1‐adrenoceptor antagonists. Out of these patients, 253 were diagnosed with type 2 diabetes. Matched with a group of 246 type 2 diabetes patients who were not diagnosed with BPH, there was no statistically significant difference of improved success rate (*P* = .06).

* *P* = .06.

Alpha 1‐adrenoceptor antagonists were the only drug class that yielded significantly better success rate in glucose control. Comparing a subgroup of patients from the above that was additionally treated with α1‐adrenoceptor antagonists (for benign prostate hyperplasia) with a matched group of patients who did not receive α1‐adrenoceptor antagonists showed a significantly higher success rates for the treated group:

61% success rate for treated group and 53% success for untreated group. *P* < .0004 and test statistic of 16.7).

Using propensity score matching, the treated group contained 1356 patients and the untreated group contained 1221 patients.

The α1‐adrenoceptor antagonists taken by the patients included alfuzocin (27%), doxazocin (18%), terazocin (6%), and tamsulosin (49%). Tamsulosin and alfuzocin are selective 1a α1‐adrenoceptor antagonists, whereas tetrazocin and doxazocin are nonselective α1‐adrenoceptor antagonists. Because of the limited sample size, we could not perform a subanalysis comparing antidiabetic potency among the different α1‐adrenoceptor agonists.

In further dividing the diabetic group according to antidiabetic drug treatment, the largest group of patients (9121) was treated with biguanides. In repeating the tests for this subgroup only, we also found a statistically significant difference in diabetes treatment success rates favoring patients treated with a1 adrenoceptor antagonists (409 treated, 380 not treated, *P* = .02, test stat. 5.23).

### 3.2 Additional analyses

1. We examined the prevalence of DM‐2 among patients concomitantly treated with drugs for BPH and found it to be 10%. Chi‐squared test for diabetes treatment success rates for patients with hypogonadism (male) did not show significant difference. (*P* = .77)
2. Chi‐squared test for diabetes treatment success rates for patients with enlarged prostate (male); Out of 26 537 patients diagnosed with enlarged prostate there were 5756 patients who did not receive α1‐adrenoceptor antagonists. Out or these patients, 253 were diagnosed with type 2 diabetes. Matched with a group of 246 type 2 diabetes patients who were not diagnosed with enlarged prostate, there was no statistically significant difference of improved success rate (*P* = .06) (Table 1).

## 4 DISCUSSION

Our analysis, using machine learning of big data, confers a significant therapeutic advantage to α1‐adrenoceptor antagonists in controlling glucose levels in DM‐2 patients receiving antidiabetic drugs. Based on animal experiments one may hypothesize that this class of drugs is involved in the regulation of basal insulin secretion^13^ resulting in the stimulation of plasma insulin levels.^14^ To examine the validity of these findings, we systematically reviewed the published literature on potential effects of this group of medications on glucose metabolism. The effects of α1‐adrenoceptor antagonists on plasma concentrations of glucose and insulin were studied in rats. Infusion of the selective α1‐adrenoceptor antagonist prazosin slightly increased glucose levels and decreased insulin concentrations.^13^ In contrast, a mouse study has shown increased basal plasma insulin levels with the α1‐adrenoceptor antagonist prazosin.^14^ After hypothetically testing data from genome wide association studies, proteomics and metabolomics, Zhang et al hypothesized that alpha 2 (but not α1‐adrenoceptor antagonists) may be favorable for glucose control in type 2 diabetes.^15^

An uncontrolled study reported on the effect of the α1‐adrenoceptor antagonist doxazocin in treating hypertensive patients with type 2 diabetes. Although there was no obvious improvement in glucose metabolism, doxazocin noticeably reduced insulin resistance.^16^ The effect of the α1‐adrenoceptor antagonist doxazocin on plasma insulin and blood glucose was studied on 10 newly diagnosed essential hypertension patients undergoing a glucose tolerance test. In addition to a lipid lowering effect over 6 months with doxazocin, there was a significant decrease in plasma insulin and blood glucose. The glucose to insulin ratio (the insulin sensitivity index) increased. The study suggested a favorable effect of doxazocin on insulin action.^17^ In a study on hypertensive patients, with or without noninsulin‐dependent diabetes mellitus, doxazocin was associated with a significant improvement in insulin‐mediated glucose disposal and lower plasma insulin, but not in diabetic patients.^18^

In mild hypertensive patients, the introduction of doxazocin was associated with significantly lower plasma insulin response to a 75‐g oral glucose load. In addition, insulin‐mediated glucose uptake was significantly greater after doxazocin.^19^ Similar results, suggesting doxazocin‐induced improvement in sensitivity to insulin, were shown by Huuponen et al.^20^ Of interest, in virtually all the above cited studies, oxazocin was given in an attempt to evaluate its antihypertensive efficacy in hypertensive patients also experiencing type 2 diabetes.

Our study is the first attempt to use machine learning, big data analytics to evaluate potential antidiabetic effects of non antidiabetic drugs taken concomitantly by diabetic patients. Out of almost 300 drug classes, only α1‐adrenoceptor antagonists conferred an antidiabetic effect after controlling for a variety of potential confounders.

It is noteworthy that almost all of our patients received the α1‐adrenoceptor antagonists for benign prostatic hyperplasia (BPH), a condition where this class of drugs is commonly used due to its proven ability to relax the smooth muscle of the urethra.^21^

Several studies have investigated the relationship between BPH and type 2 diabetes. Ozcan et al have shown a positive correlation between high prostate volume and diagnosis of diabetes mellitus in patients with BPH,^18^ a finding confirmed by Hammarsten,^22^, ^23^ while Sarma et al did not find such an association.^24^

Because α1‐adrenoceptor antagonist drugs were taken almost exclusively for benign prostate hyperplasia (BPH) in men, all other analyses were conducted on men. Hence these data do not provide sufficient information to judge whether women would also benefit from α1‐adrenoceptor antagonists, therefore, studies enrolling women to specifically test this hypotheses should be conducted.

Even by the virtue of age, it is expected that 50% of males above 60 years of age will experience BPH,^25^ of whom an estimated 10%‐20% will have type 2 diabetes.^26^ Hence the antidiabetic effects of α1‐adrenoceptor antagonists have a potentially important clinical utility to millions of men exhibiting both conditions.

Among other questions, future work will have to evaluate the relative efficacy of the different subtypes of α1‐adrenoceptor antagonists, as well as the potential risk of hypoglycemia when α1‐adrenoceptor antagonists are used among patients with BHP not suffering from type 2 diabetes.

## 5 CONCLUSIONS

Machine learning of big data is a novel method to identify effective antidiabetic effects for repurposing medications already on the market for new indications. The evidence emerging from our study, backed up by both animal and human experimental data, strongly suggest that α1‐adrenoceptor antagonists should be rigorously tested for their potential favorable impact on diabetes control through carefully designed prospective studies. Because α1‐adrenoceptor antagonists are widely used in for treating benign prostate hyperplasia at age groups exhibiting increased prevalence of type 2 diabetes, this finding is of potential clinical significance**.**

## DISCLOSURE

The authors declare no conflict of interest associated with this study. No financial support was received to conduct this study.

## AUTHORS’ CONTRIBUTIONS

GK and GN conceived the project. GK wrote the first draft. GN performed the analysis. KR oversaw the analysis. VS received permission to use the database and critically reviewed the manuscript.
